# Hydration of AMP and ATP Molecules in Aqueous Solution and Solid Films

**DOI:** 10.3390/ijms141122876

**Published:** 2013-11-20

**Authors:** Dzhigangir Faizullin, Nataliya Zakharchenko, Yuriy Zuev, Alexander Puzenko, Evgeniya Levy, Yuri Feldman

**Affiliations:** 1 Kazan Institute of Biochemistry and Biophysics, Russian Academy of Sciences, P.O. Box 30, Kazan 420111, Russia; E-Mails: natasha@mail.knc.ru (N.Z.); yufzuev@mail.ru (Y.Z.); 2 Department of Applied Physics, the Hebrew University of Jerusalem, Givat Ram, Jerusalem 91904, Israel; E-Mails: puzal@mail.huji.ac.il (A.P.); genja.levy@mail.huji.ac.il (E.L.); yurif@mail.huji.ac.il (Y.F.)

**Keywords:** dielectric spectroscopy, quartz crystal microbalance, nucleotides, bound water

## Abstract

Water enables life and plays a critical role in biology. Considered as a versatile and adaptive component of the cell, water engages a wide range of biomolecular interactions. An organism can exist and function only if its self-assembled molecular structures are hydrated. It was shown recently that switching of AMP/ATP binding to the insulin-independent glucose transporter Human Erythrocyte Glucose Transport Protein (GLUT1) may greatly influence the ratio of bulk and bound water during regulation of glucose uptake by red blood cells. In this paper, we present the results on the hydration properties of AMP/ATP obtained by means of dielectric spectroscopy in aqueous solution and for fully ionized forms in solid amorphous films with the help of gravimetric studies.

## 1. Introduction

Biomolecules are structurally complex and are greatly affected by their environment. However, the size of the surrounding water layer and the mechanism of its influence are still not clear. Like in proteins, water is an integral part of nucleic acid structures and is decisive for their stability, dynamics, and recognition [1].

Adenine nucleoside phosphates in the form of ATP, ADP, and AMP have an important role in metabolism for production, storage and transport energy. There is experimental evidence that ATP turnover during ATPase cycle of myosin motor domain is accompanied by release/uptake of hundreds of water molecules [2]. In addition to protein conformational transition, the difference in ATP and ADP hydration may contribute significantly to the balance of free and restricted water. It was suggested [3] that switching of ATP/AMP binding to the insulin-independent glucose transporter Human Erythrocyte Glucose Transport Protein (GLUT1) may greatly influence the ratio of bulk and bound water during regulation of glucose uptake by Red Blood Cells (RBC). The negatively charged ATP alters the tertiary structure of the GLUT1 by binding to the cytosolic endofacial vestibule, a process that inhibits the net sugar transport. Using dielectric spectroscopy it was shown that ATP regulated process has a significant impact on the RBC membrane interfacial polarization [4]. At elevated glucose concentrations the conformational changes of the transporter due to ATP binding are the opposite of those observed with AMP [3]. Due the high number of GLUT1 transporters in the cell membrane render, it likely those GLUT1-ATP complexes provide a significant alteration of the measured changes in the dielectric properties of the membrane [3]. Monitoring such phenomena has increasingly become of interest with the use of highly sophisticated multisensor systems that can be worn on the human body to control a variety of processes in order to derive an understanding of the metabolic state of the body [5,6]. Numerous *in vivo* and *in vitro* investigations have been carried out in order to better understand the underlying mechanisms governing the biophysical effects caused by the variation of glucose in blood for example [5]. However, the human complexity to an experimental setting can significantly impede the investigation of this mechanism, while the *in vitro* study can be well controlled.

Here, we presented the further study of AMP/ATP hydration using two completely different approaches. The study combines dielectric spectroscopy measurements of AMP/ATP aqueous solutions in the frequency band of the main water dispersion with gravimetric measurements of sorption isotherms in an attempt to elucidate the composition of water-AMP and water-ATP complexes in solid films. The findings will be relevant to contribute to the clarification of underlying mechanisms and their biophysical impacts *in vivo*.

**Figure 1:**
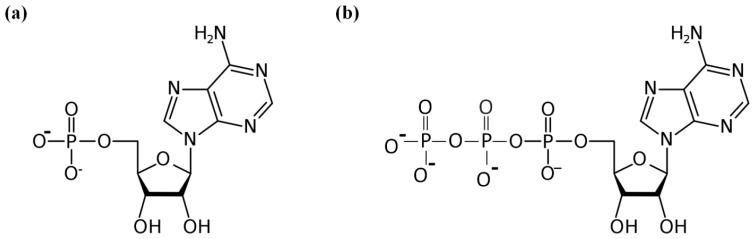
(**a**) AMP^2−^ molecule at pH 8. And (**b**) ATP^4−^ molecule at pH 8.

The considered nucleotides: ATP and AMP nucleotides have relatively large dipole moments. The measured value of the ATP dipole moment in aqueous solution is ~230 D. The estimated value of the dipole moment of AMP is significantly smaller ~45 D [3]. At the same time both AMP and ATP (see Figure 1) become highly charged ions in aqueous solution. Therefore following our main hypothesis, namely water is considered as the dipole subsystem, and the solute molecules play the role of the matrix, in the ATP/AMP aqueous solutions we have two types of dipole-matrix interactions that allow us to evaluate the number of water molecules interacted with the nucleotide in solution. In the case of dielectric measurements of AMP/ATP aqueous solutions we are going to extend the phenomenological approach [7] that was recently applied to water behavior in ionic and nonionic solutions [8–10]. It was shown that whenever water interacts with another dipolar or charged entity in aqueous solutions, the resulting high-frequency spectrum displays a Cole–Cole (CC) behavior [11]:

(1) $$ɛ*(\omega )={ɛ}_{\infty }+\frac{\Delta ɛ}{1+{\left(i\omega \tau \right)}^{\alpha }}$$

where *i*^2^ = −1, ω = 2π*ν* is the frequency, ɛ*_s_* and ɛ*_∞_* are the extrapolated low-frequency and high-frequency permittivity and Δɛ = ɛ*_s_* − ɛ*_∞_* dielectric strength; τ is the relaxation time, and the exponent α (0 < α ≤ 1) is referred to as a measure of the dielectric losses relaxation peak broadening. The nature of the coupling between the number of the relaxation acts occurring during the characteristic relaxation time and the molecular structure, in which they occur, still remains unclear. One may elucidate this coupling further by exploiting the unutilized parameters Δɛ, ɛ*_s_*, and ɛ*_∞_* of Equation (1). These parameters are related to structural aspects of the relaxation and Froehlich pioneered this approach by associating them with the number of dipoles involved [12]. Using the general relationship for polar dielectrics, he introduced a new function that described the temperature behavior of the polarization of the system under consideration:

(2) $$B=\Delta ɛ\frac{2{ɛ}_{s}+{ɛ}_{\infty }}{3{ɛ}_{s}}T=\frac{1}{3{ɛ}_{0}kV}\langle M^{2}\rangle$$

Here *k* is the Boltzmann constant, *T* is the absolute temperature, *V* is a volume with *N* microscopic cells containing some dipoles or charges, respectively, ɛ*_0_=* 8.85 10^−12^*F/m*. The electric dipole moment of this volume is defined as $M=\sum_{i=1}^{N}m_{i}$, with *m_i_* as the average dipole moment of the *i*-th cell, and the brackets <…> in Equation (2) indicating a statistical averaging over all possible cell configurations. The dipole moment of the macroscopic volume of material polarized by one cell with the dipole moment **m** is denoted by m^*^ As the same underlying thermodynamic variables (temperature, pressure, concentration, *etc.*) drive *B* as well as *x*, it appears to be obvious to consider a relationship between the two parameters. The use of this function for analysis of the dielectric strength of the main water relaxation process allows one to estimate the effective number of the water molecules associated with one nucleotide, as it was shown previously [10].

Besides the dielectric approach, attempts to be considered in establishing the detailed balance of hydration should also take into account the difference in water bound by adenosine nucleoside phosphates in specific conformations. The main issue in solution is that hydration water is often difficult to distinguish from the bulk one. A number of crystallographic and spectroscopic studies reveal highly specific structural transitions to occur on the hydration-dehydration of nucleoside and nucleotide crystals [13–15]. Crystallographic studies of humidity induced phase transitions give information on atomic resolution but are limited to relatively rear cases where transition proceeds retaining the single-crystal state. So, no information has been obtained regarding the structure of water-nucleotide complexes in fully ionized forms of AMP^2−^ and ATP^4−^, which avoid crystallization [16]. Nevertheless, the knowledge of possible hydration-mediated structure transitions of these nucleotide species is in demand because of their physiological relevance. In this work we attempted to elucidate the composition of water-AMP and water-ATP complexes in solid films measuring sorption isotherms gravimetrically. We employed the quartz crystal microbalance technique to measure both the mass of water uptake and the value of mechanical loss, which is sensitive to film viscosity [17]. Comparative analysis of these uncoupled features allowed us to determine the number of distinct hydrates as well as the amount of water molecules constituting them in these films.

In this paper, we combined two independent methods such as the dielectric spectroscopy of nucleotides aqueous solutions and the quartz crystal microbalance technique nucleotides films in order to compare the levels of ATP/AMP hydration.

## 2. Results and Discussion

### 2.1. Dielectric Spectroscopy

**Figure 2:**
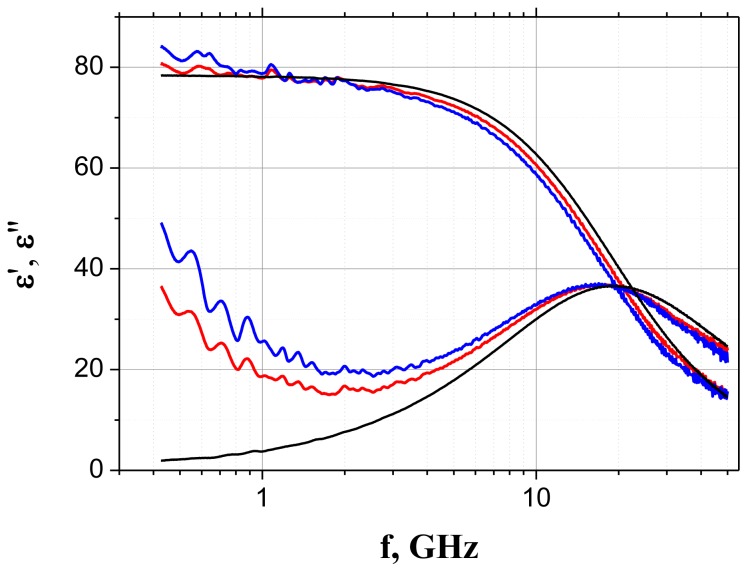
The real part ɛ′(*f*) and the imaginary part ɛ″(*f*) of the dielectric spectra (1021 frequency points) for water (black line), and for 0.06 mol/L aqueous solutions of AMP (red line) and ATP (blue line) at 25 °C. Reprinted with permission from reference [10], (Copyright 2012, American Institute of Physics).

Typical dielectric spectra in the frequency band 0.5–50 GHz for both of AMP and ATP aqueous solutions together with the spectra of the solvent are presented in Figure 2. Similar systematic difference between the spectra of pure water and the solutions are observed for all other concentrations. In this frequency band, the observed spectra show the single relaxation process that reflects the water property variations at different concentrations.

The experimental spectra were fitted using a sum of a CC function [Equation (1)] and additional conductivity term –*i*σ/(ɛ_0_ω), where σ is the electrical *dc* conductivity. An in-house designed program Datama [18] was used for the fitting of both real and imaginary parts of the complex dielectric permittivity simultaneously. The values of the fitting parameters are presented in the paper [10].

**Figure 3:**
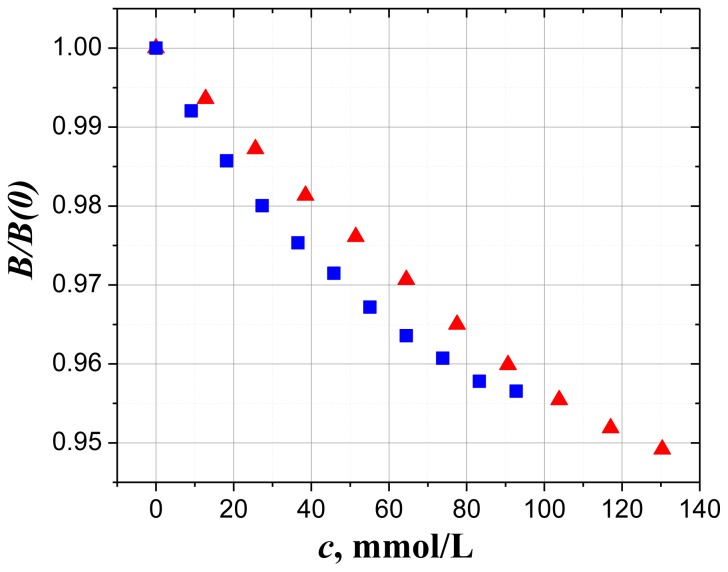
The concentration dependence of the normalized Froehlich function *B*/*B*(0) for aqueous solutions of: AMP (red triangles) and ATP (blue squares) at 25 °C. Reprinted with permission from reference [10], (Copyright 2012, American Institute of Physics).

Aqueous solutions of AMP and ATP are typical examples of complex molecules with the dual nature of water-matrix interaction. To clarify the meaning of this duality let us consider the concentration dependence of the normalized Froehlich function *B*(*c*)/*B*(0), shown in Figure 3. Here *B*(*c*) and *B*(0) are the Froehlich functions for solutions at different concentration and for pure water (solvent) respectively. Both AMP and ATP solutions demonstrate non-linear decay of Froehlich’s function with concentration, and the ATP curve shows more pronounced deviation from the linear law. The smooth monotonic decay for both nucleotides indicates the absence of any dramatic changes in the structure, and it gives us the opportunity to suggest that around each solute molecule a shell of water molecules is formed. Note that the separation of water molecules in the vicinity of the solute: the water that belongs to the shell and the next following layers of perturbed water. This separation is very uncertain. The only requirement for this separation is the constant number of water molecules in the shell at diluted solute concentration.

The Froehlich’s function *B* can be calculated using the experimental data or can be evaluated in terms of the macroscopic dipole moment ***M*** [Equation (2)]. If we extend the approach developed previously [8,9], we should consider the following model as the sum of three random components of the total dipole moment:

(3) $$M=\sum_{i=1}^{N_{W}}\mu_{wi}+\sum_{f=0}^{N^{+}}M_{f}^{+}(1-\delta_{f0})\sum_{l=0}^{N_{a}}M_{l}^{a}(1-\delta_{l0})$$

where μ*_w_* = 3.84 D is the dipole moment of water molecules in the liquid state [8], *N_w_* and *N_a_* are the corresponding numbers of water and nucleotide molecules in the volume *V. N*^+^, $M_{f}^{+}$ are the numbers of ions and the dipole moments of the water clusters of hydration shells in the vicinity of the positive ions, and δ*_ik_* is the Kronecker symbol. Since we are working with AMP and ATP disodium salts each solute molecule contains a nucleotide molecule and two molecules of sodium. Therefore, with the dissociation of each molecule, the two sodium ions are formed *i.e*., *N*^+^ = 2*N_a_* [19]. Each ion shell contains the specified number of water molecules, as was mention above.

The Equation (3) is more convenient to rewrite in the normalized form. Dividing it by an average square dipole moment of the bulk water $\langle M^{2}(0)\rangle ≅N_{w}(0)\mu_{w}^{2}g_{w}$ (at *c* = 0), and taking into account its relationship with *B* function [Equation (2)] we can get the effective total number of perturbed water molecules per solute molecule as we presented it earlier[10]:

(4) $$N_{cl}=\left|\left[\frac{B}{B(0)}\frac{N_{w}(0)}{N_{w}}-1\right]\frac{N_{w}g_{w}}{N_{a}}-4N_{w}^{+}(1+g_{w})\right|$$

where *g_w_* is the Kirkwood correlation factor for pure water, that is equal 7/3 [8], $N_{w}^{+}$ is the average number of water molecules in the sodium ions hydrated shells (we know it from previous work [9]).

**Figure 4:**
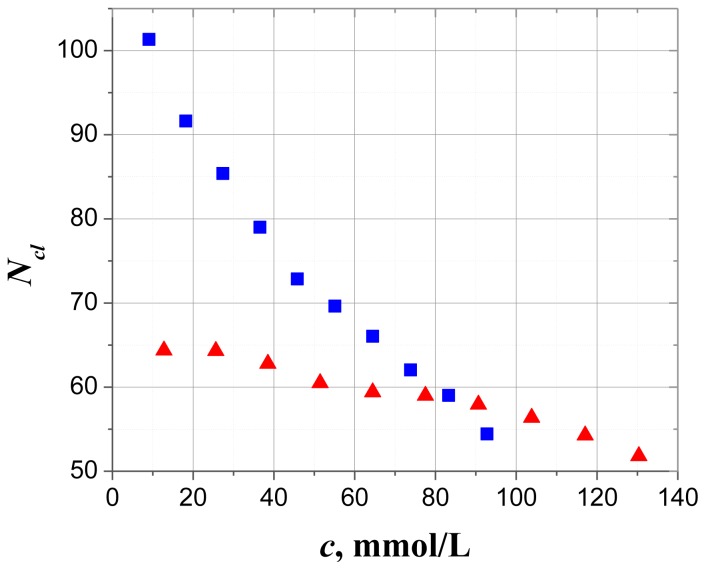
The concentration dependence of the effective number of water molecules in the shell around AMP (red triangles) and ATP (blue squares) molecule, formed by the dipole component and by its negative charges at 25 °C. Reprinted with permission from reference [10], (Copyright 2012, American Institute of Physics).

The results of *N_cl_* calculations with the help of Equation (4) are presented in Figure 4 and demonstrate the decay of the effective number of water molecules bound to the AMP/ATP molecules with their concentration. It is clear from Equation (4) that this decay for both solutes is determined mainly by the factor *N_w_/N_a_* (*i.e.*, by the sharp drop in the total number of water molecules per nucleotide).

### 2.2. Gravimetry

#### 2.2.1. Water Sorption

**Figure 5:**
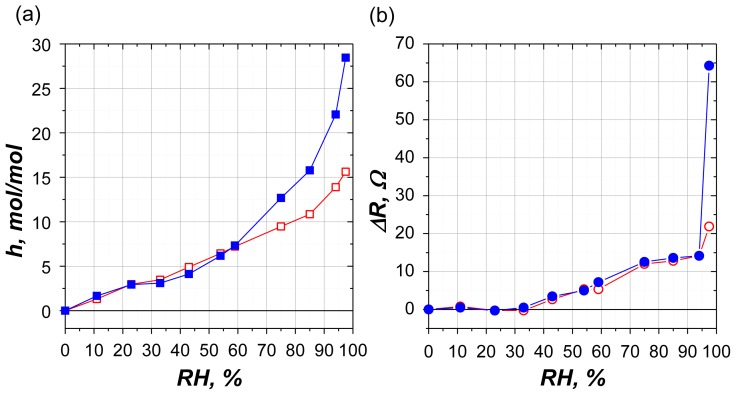
(**a**) Sorption isotherms in moles H_2_O per mole of nucleotide, of AMP (empty squares) and ATP (filled squares) solid films; And (**b**) Equivalent loss resistance in AMP (empty circles) and ATP (filled circles) solid films.

Figure 5a displays molar isotherms of water vapor sorption on solid ATP and AMP films. Parameter *h* for both chemicals increases with the rise of humidity, slowly at first and more steeply above 80%–85%. Isotherms nearly coincide up to 60% relative humidity and diverge above that RH. The region of coincidence corresponds to the sorption of up to 7 mol of water per mole of nucleotide. Above RH 60% water sorption on the ATP film exceeds that for AMP. Just above RH 60% in amorphous nucleotide samples the sorption of weakly bound liquid-like water perceptible [20]. It follows from Figure 5a that ATP molecule binds significantly more water of this type than AMP does.

#### 2.2.2. Mechanical Losses

Sorption of water by nucleotide film changes its mechanical properties, which is reflected in magnitude of energy dissipation in the sample upon excitation of mechanical vibrations. Figure 5b shows the dependence of equivalent loss resistance Δ*R* on relative humidity of atmosphere. The obtained dependences for ATP and AMP samples are very close and have a stepwise form: upon increasing of RH the growing parts alternate by the flat ones, where the losses are independent on humidity. In RH range 0%–30% hydration-induced losses in both samples are constant and close to zero despite the sorption of 3 mol of water per mole of nucleotide. Hardness and elasticity of hydrated AMP and ATP films do not differ from those for dry films. Above RH 30%, ΔR increases gradually reaching next plateau at RH 75%–95%, where Δ*R* value remains constant despite the continued increase in water sorption. Δ*R* rises steeply only for values above RH 95%. The outlined stepwise behavior of mechanical losses mismatches the sorption data but reflects the stepwise changes in internal structure of the nucleotide film during humidification.

#### 2.2.3. Sorption Centers

From crystallographic studies it is known that the hydration of phosphonucleosides is occurring in the form of discrete hydrates [21]. The number of structurally different hydrates as well as the number of water molecules involved in their formation depends on the relative humidity of atmosphere, the chemical nature of the nucleoside residue, the number of phosphate residues and degree of their ionization and the type of counter ion [21,22]. Each form of hydrates is stable in particular RH range and is characterized by a specific spatial arrangement of nucleotide groups and water molecules. Detailed structure of crystalline hydrates of ATP^2−^ was examined by X-ray diffraction and formation of hydrates with one, two and three water molecules coordinated with the Na^+^ ions, negatively charged oxygen atoms of phosphate groups and polar atoms of the nucleoside and ribose has been revealed [14]. Unfortunately, there are no crystallographic data in literature on hydrates of fully ionized species ATP^4−^ and AMP^2−^ most probably due to their inability of crystallization [16]. There are water sorption data for polycrystalline samples of several adenosine phosphates, namely for Na_2_ATP, Na_3_ADP and NaAMP [21], but not for Na_4_ATP and Na_2_AMP. This is the reason that in this study we are limited to compare our results with the above cited hydration data.

Comparison of ATP and AMP hydration data revealed the coincidence of molar isotherms in the RH range 0%–30% (Figure 5a). It means that in spite of different molecular weight both chemicals exhibit equal number of identical sorption centers. Chemical structure of both nucleotides differs in the number of phosphate residues only. Therefore, in this RH range, two additional phosphate groups of ATP are inaccessible for water molecules while the remainder forms a hydration complex similar to the composition of AMP hydrate. RH range embracing the first plateau on the ATP and AMP isotherms (Figure 5a) includes the RH domain, in which the formation of crystalline hydrate Na_2_ATP·2H_2_O was observed upon vapor sorption on Na_2_ATP crystals [13,15]. Another sorption plateau could be disclosed as a knee on ATP isotherm in the range 75%–85% RH (Figure 5a), where the formation of Na_2_ATP·3H_2_O crystalline hydrates was observed early [13,15].

It means that, despite the differences in physical structure and degree of ionization, the RH ranges corresponding to formation and stabilization of discrete hydrates are the same in both solid films and crystals.

#### 2.2.4. Conformational Transitions and Structure of Solid Film

Formation of discrete hydrates of certain composition is coupled with conformational transitions in nucleotide molecules and occurs in relatively narrow RH ranges. Humidity-dependent reversible structural transitions of various mono- and polycrystalline nucleotide samples have been successively examined [13,23]. Clear step-like character of water sorption by nucleotide crystals is a consequence of highly cooperative conformational transitions. Figure 5 depicts that water sorption by nucleotide films proceeds also in the stepwise manner, although transitions look fuzzier. The question arises about the origin of smoothed shape of sorption isotherms: could it be associated with the simultaneous presence of crystalline and amorphous regions with different mechanisms of water sorption?

We believe that analyzing mechanical properties of the films could help to answer this question. The origin of mechanical losses, Δ*R*, is mainly associated with the film structure but it is not directly coupled with the level of water sorption [17]. Based on this property one could recognize that the RH ranges where Δ*R* is independent on relative humidity (Figure 5) correspond to the unchanged structure of the sample. There are two such ranges and both correspond to the same RH intervals, namely 0%–33% and 75%–94%, where the plateaus are discerned on the sorption isotherms, corresponding to hydrates of different composition. The stepwise increase of mechanical losses accompanying by water sorption conforms the formation of higher order hydrates, which contain more water molecules and, consequently, have less rigid structure.

Following this reason it is possible to deduce that the spatial structure of the films is uniform and changes in stepwise manner as all-or-none process at characteristic transition ranges, *i.e.*, the assumption of the coexistence of crystalline and amorphous regions is not justified.

Steep increase of losses Δ*R* above RH 94% may reflect an increase of the film viscosity due to its swelling upon sorption of weakly bound water, which is not included in the structure-forming hydrates.

#### 2.2.5. Model of Hydration

**Figure 6:**
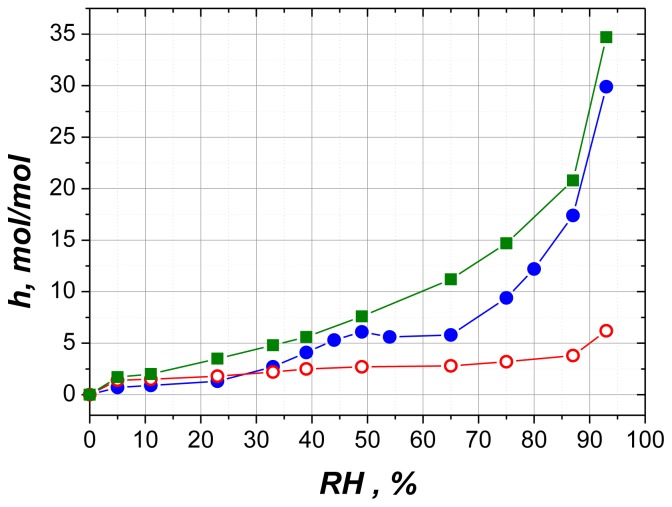
Water sorption on polycrystalline samples of NaAMP (empty circles), Na_2_ATP (filled circles) and Na_3_ADP (squares). All data are reproduced from reference [21].

The obvious contradiction still remains between the discrete character of formed hydrates and smoothness of sorption curve. To resolve this disparity it is advisable to outline factors governing the form of sorption isotherms for nucleotides. Figure 6 compares hydration of polycrystalline samples of Na_2_ATP, Na_3_ADP and NaAMP [21]. All three compounds are sodium salts of adenosine-5′-phosphoric acid, so the effects of the nucleoside and the counter ion are excluded. These compounds differ only by the number of phosphate groups and the degree of ionization. From Figure 6 it could be deduced that the degree of ionization of phosphate groups has the most prominent influence on the shape of the isotherms and sorption level. Phosphates are ionized in two stages (p*K* 1 and 6.5–7)—at low pH each group except the terminal one losses a proton and at pH > 7 the last proton is removed from the terminal phosphate. Among the compounds presented in Figure 6, Na_3_ADP is the only one ionized completely; it sorbs more water than two other chemicals and at the same time its isotherm has mostly smoothed and featureless shape.

As it has been stated above, the hydration of films from fully ionized ATP and AMP proceeds through formation of discrete hydrates in the same RH ranges as it does in crystals of partially ionized nucleotides. It is reasonable to deduce that in Na_2_AMP, Na_4_ATP as well as in Na_3_ADP the last ionizable oxygen of terminal phosphate is not involved in the formation of discrete hydrate and its hydration proceeds independently and continuously.

**Figure 7:**
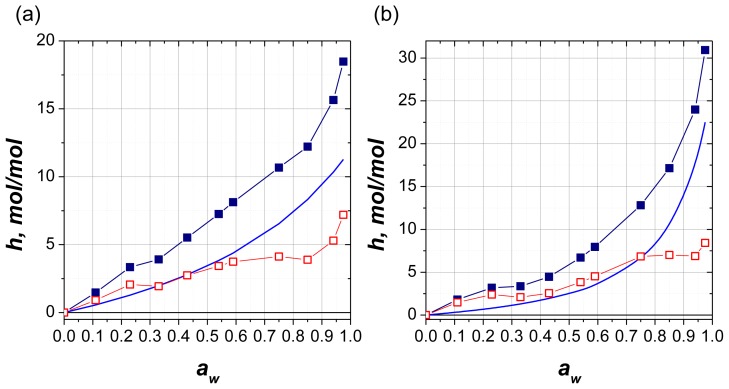
(**a**) Experimental isotherm of water sorption on AMP (filled squares), the calculated isotherm following Flory-Huggins model (solid line) and the difference between the experimental and theoretical dependencies (empty squares). And (**b**) The same data for ATP.

Based on this assumption, we attempted to decompose experimental isotherm into two components, discrete hydrates and continuous water sorption on ionized oxygen of terminal group. It is known that the ionic groups in solid samples and in solution are hydrated by forming hydration shell from rotationally hindered water molecules with its size dependent on water activity. We approximate the ionic hydration in films by third term in D’Arcy–Watt equation, which describes the cluster sorption using the Flory–Huggins solution model [24]:

(5) $$h=\frac{K_{4}\cdot K_{5}\cdot x}{1-K_{5}\cdot x}$$

where *K*_4_ and *K*_5_ are parameters and *x* is the water activity in solution. Assuming that under the equilibrium water activity in the gas phase is equal to that in solid solution, *x = a_w_*. The parameters were adjusted to give after subtraction of model function from experimental data a stepped curve with a clear plateau (Figure 7). Parameters of fitting model and the values of hydration corresponding to plateau regions are shown in Table 1.

#### 2.2.6. Comparison with Solution

The Table 1 reveals that with increasing number of charges of phosphate groups in nucleotides (Figure 1) there is an increase in the amount of the strongly bound in a structure-forming hydrate (*h*_2_) and weakly bound (*h*_3_) water. Obviously, *h*_3_ value reflects hydration not only of the terminal phosphate, but also of the second hydration layer of other phosphate groups, perceptible only at high RHs. In the case of solutions the role of ion-dipole interactions is increased with increase of nucleotide concentration. Moreover, they prevailed at the limit of highly concentrated solutions or hydrated solid films. As we expected, an initial concentration point [10] corresponds to the upper physiological limit of ATP and in this case, the effective number of water molecules *N_cl_* bounded to ATP molecule is ~1.6 times greater than for AMP molecule. This result explained due to the large permanent dipole moment and the total charge of the ATP molecule compared to the AMP one and is in good agreement with the ratio obtained for films. The authors of the preceding study [25] described the same phenomena in terms the two types of bound water (hyper-mobile and constrained) assigning each to the separate contribution into the observed dielectric spectra. In spite of the fact that these approaches are different, the results still can be used for comparison of the ratios between the total number of bound water molecules in case of nucleotide films and solutions. The latter are given in Table 2.

Indeed it follows from Table 2 that ATP compared to AMP binds significantly more water in solution and in solid state, moreover it not depends on the amount of environmental water.

## 3. Experimental Section

ATP (Sigma, A7699, adenosine-5′-triphosphate disodium salt hydrate; ≥99%) and AMP (Fluka, 01930, adenosine-5′-monophosphate disodium salt; ≥99.0%) were purchased from Sigma–Aldrich Israel Ltd. (Rehovot, Israel) and used without further purification.

### 3.1. Dielectric Spectroscopy

The solutions were prepared in triple distilled water in steps of 0.5 mass percent in the concentration range from 0.5 mass percent to 5 mass percent using a precision balance (0.1 mg). The percentage was chosen up to a saturation of the ATP solution [19]. All samples were freshly prepared just before the experiment. Dielectric measurements were carried out in the frequency range from 500 MHz to 50 GHz (1021 frequency points in log-scale) using a Microwave Network Analyzer (Agilent N5245A PNA-X; Agilent Technologies, Santa Clara, CA, USA) and an Agilent slim-form probe (85070E) (Agilent Technologies, Santa Clara, CA, USA). A special stand for the slim-form open ended coaxial probe was designed and combined with a sample cell for the liquids (total volume of ~7.8 mL). The cell was contained inside a thermal jacket and attached to a Julabo CF 41 heat circulatory system (JULABO GmbH, Seelbach, Germany), based on oil. The cell was held at 25 °C by the circulator-thermostat with temperature fluctuations less than 0.1 °C. The calibration of the system was performed with the aid of three standards: open coaxial line, the Agilent standard short circuit and reference sample (pure water at 25 °C). The whole measuring system was placed in an air-conditioned room maintained at 25 ± 1 °C. Each solution was measured at least four times in succession. The real and imaginary parts ɛ′(ω) and ɛ″(ω) were evaluated using the Agilent Materials Measurement Software 85070 (Agilent Technologies, Santa Clara, CA, USA) with accuracy Δɛ′/ɛ′ = 0.05, Δɛ″/ɛ″ = 0.05 [26]. In addition the densities of the solutions were measured at the same temperature using a DMA 5000 density meter (Anton Paar, Graz, Austria).

### 3.2. Gravimetry

Exactly 1.5 mg/mL solutions were prepared using double distilled water. pH of AMP solutions was 7.9, which corresponds to complete ionization of the molecule up to AMP^2−^ (Figure 1a). ATP being dissolved in water gave pH 4 corresponding to its partial ionization up to ATP^2−^. By adding NaOH this solution was adjusted to pH 7.9, giving complete ionization of ATP up to ATP^4−^ (Figure 1b). The films were prepared by depositing approximately 2 μL solution on silver electrodes on both sides of quartz crystal element, spreading and drying. The mass of the film on each electrode was 3–5 μg. The film-coated sensor was placed in a glass flow-through cell and blown by stream of humid air. Relative humidity (RH) of air was controlled by slow bubbling through saturated salt solutions. It is assumed that water activity (*a_w_*) in saturated salt solution is equal to that in vapor phase and RH = *a_w_* × 100%. Air dried over P_2_O_5_ was taken as zero RH. The temperature was maintained at 22 ± 1 °C using the water bath. Mass of the film and water uptake were measured using home-made 8 MHz quartz crystal oscillator with energy loss monitoring. Oscillator frequency variation is proportional to mass change of the sample deposited on the sensor electrodes [17].

Hydration value in molar units was calculated as follows:

(6) $$h=\frac{\Delta f}{\Delta f_{0}}\frac{M_{nucleotide}}{M_{water}}$$

where *h* is mol of water/mol of nucleotide; Δ*f* = *f_h_* − *f_m_*, Δ*f*_0_*= f_m_* − *f*_0_, *f*_0_ is the frequency of unloaded crystal oscillator, *f_m_* is the frequency of oscillator loaded with dry sample, and *f_h_* is the same with hydrated sample, *M* is molar mass.

In addition, the value of loss resistance *R* in equivalent oscillatory circuit was measured. It is proportional to the mechanical losses imposed by the sample on quartz crystal oscillations. The *R* value was measured after saturation of sorption, so it should be considered equilibrium one. Losses due to hydration were calculated using the formula:

(7) $$\Delta R=R_{h}-R_{m}$$

where *R_m_* is the losses in a dry sample, and *R_h_* is the same in a hydrated sample.

Inaccuracy of measurements was determined by averaging the repeated hydration and drying process for samples at RH 59% and 0%, respectively. The errors did not exceed 5% for *h*, and 10% for Δ*R* values.

## 4. Conclusions

Hydration properties of AMP and ATP have been studied by the gravimetric sorption method. It was found that in the amorphous solid films the AMP^2−^ and ATP^4−^, hydration proceeds through the formation of discrete hydrates formed by structurally bound water molecules. Unlike the case for partially ionized nucleotides, hydration of their completely ionized forms revealed a significant contribution from unstructured water. This is continuously sorbed by the charged oxygen of the terminal phosphate. ATP sorbs two times more water than AMP. The obtained numbers of bound water molecules agrees with those found in dilute aqueous solutions, studied by dielectric spectroscopy. The effective number of water molecules bound by the solute increases with concentration. However, a large amount of bulk water is still available. The relatively large numbers of water molecules bounded by AMP and ATP, along with the significant difference between them, proves the hypothesis that metabolic processes may manifest themselves through the influence of the balance between bound and bulk water.

These findings further contribute to the understanding of the fundamental mechanisms related to glucose metabolism, their biophysical implications and help to explain the sensitivity of the *in vivo* measurements of glucose variation in blood [10].

## Figures and Tables

**Table 1:** Decomposition of the experimental isotherms for AMP and ATP: *K_4_* and *K_5_* are parameters of Flory–Huggins model, *h_1_* and *h_2_* are the hydration levels at first and second plateaus, respectively, *h*_3_ is the hydration at *a_w_* = 0.975 from the Flory–Huggins model. Values of *h* are expressed in moles of water per mole of nucleotide.

Nucleotide	*K_4_*	*K_5_*	*h_1_*, mol H_2_O/mol nucleotide	*h_2_*, mol H_2_O/mol nucleotide	*h_3_*, mol H_2_O/mol nucleotide
AMP^2−^	8	0.6	2 ± 0.2	4 ± 1	11 ± 3
ATP^4−^	3	0.905	2 ± 0.2	7 ± 1	22 ± 5

**Table 2:** Ratio values of bound water in solid films of AMP and ATP and in solution (^a^this study) and (^b^from [25]).

Sample state	Ratio values of bound water
Solid films *h*_2 ATP_/*h*_2 AMP_	1.8
Solid films *h*_3 ATP_/*h*_3 AMP_	2
^a^Solution *h*_ATP_/*h*_AMP_	1.6
^b^Solution *h*_ATP_/*h*_AMP_	2.1
